# Feeding of soy protein isolate to rats during pregnancy and lactation suppresses formation of aberrant crypt foci in their progeny's colons: interaction of diet with fetal alcohol exposure

**DOI:** 10.1186/1477-3163-3-14

**Published:** 2004-10-15

**Authors:** Amanda L Linz, Rijin Xiao, James G Parker, Pippa M Simpson, Thomas M Badger, Frank A Simmen

**Affiliations:** 1 Arkansas Children's Nutrition Center, 1120 Marshall Street, Little Rock, Arkansas, 72202, USA; 2 Department of Physiology and Biophysics, University of Arkansas for Medical Sciences, Little Rock, Arkansas, 72202, USA; 3 Department of Pediatrics, University of Arkansas for Medical Sciences, Little Rock, Arkansas, 72202, USA

## Abstract

Soy protein isolate (SPI) in the diet may inhibit colon tumorigenesis. We examined azoxymethane (AOM)-induced aberrant crypt foci (ACF) in male rats in relation to lifetime, pre-weaning, or post-weaning dietary exposure to SPI and also within the context of fetal alcohol exposure. Pregnant Sprague Dawley rats were fed AIN-93G diets containing casein (20%, the control diet) or SPI (20%) as the sole protein source starting on gestation day 4 (GD 4). Progeny were weaned on postnatal day (PND) 21 to the same diet as their dams and were fed this diet until termination of the experiment at PND 138. Rats received AOM on PND 89 and 96. Lifetime (GD 4 to PND 138) feeding of SPI led to reduced frequency of ACF with 4 or more crypts in the distal colon. Progeny of dams fed SPI only during pregnancy and lactation or progeny fed SPI only after weaning exhibited similarly reduced frequency of large ACF in distal colon. Number of epithelial cells, in the distal colon, undergoing apoptosis was unaffected by diet. SPI reduced weight gain and adiposity, but these were not correlated with fewer numbers of large ACF. Lifetime SPI exposure similarly inhibited development of large ACF in Sprague Dawley rats whose dams were exposed to ethanol during pregnancy. In summary, feeding of SPI to rat dams during pregnancy and lactation suppresses numbers of large ACF in their progeny, implying a long-term or permanent change elicited by the maternal diet. Moreover, results support the use of ACF as an intermediate endpoint for elucidating effects of SPI and its biochemical constituents in colon cancer prevention in rats.

## Background

Colorectal cancer is the third leading cause of cancer-related deaths in the U.S.; an estimated 147,500 new cases of this disease will have occurred in the U.S. during 2003 [1]. Globally, this cancer is a significant health problem as its incidence is increasing with the burgeoning of the aged population and with 'Westernization' of diets. Most recently, the cancer incidence rates for the ascending colon have increased whereas those for all other colon subsites (transverse, descending, sigmoid and rectum) have stabilized or declined slightly [2,3]. The incidence of colorectal cancer, like that for breast and prostate cancers, is lower in Asian than American and European populations [4]. This has implied a possible protective effect of the Asian diet and of soy foods in particular, on colon cancer incidence. Epidemiological studies are generally supportive of the postulated protection against colorectal cancer incidence by consumption of soy components and soy foods [4-6]. However, these studies were not designed to decipher possible effects of lifetime vs. developmental stage-specific dietary exposure to soy. In this regard, soy consumption during adolescence may preferentially reduce subsequent risks for breast cancer in later adults [7,8].

Soy foods are the main dietary sources of isoflavones, which are implicated in cancer prevention [4,5,9]. Soy foods are a source of other potentially cancer-preventive substances as well, including saponins, protease inhibitors, and other bio-active peptides and proteins [9-13]. However, the literature concerning effects of pure isoflavones or processed soy products (soy flour, soy protein) in animal models of colon cancer is mixed. Many investigators utilized the rat and administered a chemical carcinogen, either dimethylhydrazine (DMH) or azoxymethane (AOM). Both agents rapidly induce formation of aberrant crypt foci (ACF) and subsequent development of colon adenomas and adenocarcinomas. Investigators have utilized ACF number and type, tumor number and type, or the combination, as endpoints to examine effects of soy and soy constituents on colon cancer. Dietary genistein (the predominant soy isoflavone) was inhibitory to ACF formation in AOM-treated rats [14,15]. Order of protection (measured as ACF number) was genistein > defatted soy flour > full-fat soy flakes > soy concentrate (isoflavone-depleted) [16]. Similarly, soy bean saponins inhibited ACF incidence in AOM-treated mice at 14 weeks post-initiation [11]. However, in the study of Gee *et al*., [17], feeding of isoflavone-containing soy protein isolate or of genistein (with casein) for 7 days prior to DMH treatment (and switching to casein diet thereafter) actually promoted ACF numbers in the distal colon; whereas, feeding of these same diets for 42 days immediately after carcinogen administration had no effect. Davies *et al*. [18] formulated diets to mimic the Western type (i.e., high fat, low calcium) diet supplemented with low or high isoflavone-containing soy protein isolates, and ACF and tumors were subsequently measured in AOM-rats. Increased numbers of small ACFs were found at 12 weeks, post-carcinogen administration in the isoflavone-enhanced group. Therefore, there is a lack of consensus for effects of soy or soy components on chemically-induced ACFs in rodents and the underlying basis of these discrepancies is unknown.

The picture is also unclear when tumors rather than ACFs are used as the end point. In an early study, soybean protein did not differ from beef protein in terms of relative numbers of colon tumors in DMH-treated rats [19]. Defatted soybean meal was not protective in the same model [20]. However, we previously reported the protection afforded by lifetime-feeding of a soy protein isolate, against colon carcinoma in AOM-treated male Sprague Dawley rats [21]. Dietary genistein, on the other hand, had no effect on colon adenocarcinoma incidence or multiplicity of invasive colon carcinoma, yet actually increased noninvasive and total adenocarcinoma multiplicity [22]. In contrast, soy protein isolates with two levels of total isoflavones did not elicit differences in colon tumorigenesis in the Min mouse model of intestinal cancer [23]. However, feeding of a high molecular weight insoluble fraction from proteinase-treated soybean protein isolate suppressed colon tumor numbers in rats [24,25].

The above studies used diets made with soy protein isolates or soy components prepared in different ways and supplemented to varying levels. Furthermore, diets usually were fed just prior to or concurrent with the chemical carcinogen in order to focus on initiation or progression of tumorigenesis. Some studies did not account for the fact that commercial rodent diets can include soy protein; moreover there is significant maternal-fetal transfer of isoflavones in rats fed soy-containing diets [26]. Thus, pre-exposure to soy constituents during various stages of an animal's life cycle potentially complicates interpretation of the reported results. With the single exception of the study from our group [21], no studies examined the effects of lifetime (including gestational) exposure, by feeding of soy or purified soy components, on colon tumor or ACF incidence. In view of the significant use of soy-based infant formulae, which accounts for greater than 25% of the infant formula currently sold in the United States [9,27], it is important to examine potential SPI effects on colon tissue pre-disposition to cancer. Here, we utilize male Sprague Dawley rats fed AIN-96G diets formulated with casein or SPI to examine dietary effects on AOM-induced ACF incidence and multiplicity. We also examine effects of dietary SPI at pre- and post-weaning vs. lifetime consumption. Lastly, we confirm the SPI effect on colon ACF biogenesis in a second model, namely, AOM-treated rats whose dams were exposed to ethanol during their pregnancy.

## Methods

### Solid Diets

Diets contained either casein or SPI as the sole protein source (200 g/Kg diet) and their formulation has been previously described [21,28]. Casein (ALACID 741) was from New Zealand Milk Products (North America) Inc. (Santa Rosa, CA). SPI was a gift from DuPont Protein Technologies (St. Louis, MO). Total isoflavone content was 3.70–3.98 mg/g protein and total aglycone equivalents were 2.13–2.32 mg/g protein for the SPI. Corn oil replaced soybean oil and essential amino acid content was maintained at levels for that of the AIN-93G diet [29]. Diets were prepared by Harlan Teklad (Madison, WI).

### Animals

Animals were housed in an AAALAC-approved animal facility at the Arkansas Children's Hospital Research Institute; animal use protocols were approved by the University of Arkansas for Medical Sciences Institutional Animal Care and Use Committee. Animals were housed in polycarbonate cages and allowed *ad libitum *access to diet and water. Animal rooms had constant humidity and a 12-h light-dark cycle.

### Expt. I. Lifetime, Pre-weaning and Post-weaning Diets

Pregnant Sprague Dawley dams from Harlan, Inc. (Indianapolis, IN) were received at gestation day (GD) 4 and immediately assigned in random fashion to casein or SPI diet. At postnatal day (PND) 2, each litter was culled to 5 males and 5 females (females were used in an unrelated experiment). At weaning, animals were divided into four diet groups: lifetime (GD 4 to PND 138) casein, n = 25; lifetime SPI, n = 25; casein to SPI, n = 25; and SPI to casein, n = 25. Diet switchovers were performed at PND 21 and all rats were given AOM on PND 89 and 96. Rats were injected subcutaneously with AOM (Midwest Research Institute) in saline, 15 mg/kg body weight. Animals were weighed weekly from birth and were euthanized at PND 138 for ACF determination or TUNEL assay.

### Expt. II. Intra-gastric Infusion of Ethanol-containing CAS and SPI Liquid Diets during Pregnancy

Pregnant rats at GD 5 were surgically implanted with an intra-gastric cannula as previously described [30]. Dams were fed casein plus ethanol (n = 5) or SPI plus ethanol (n = 7) by total enteral nutrition (TEN) from GD 6 – GD 19 [30]. TEN diets were isocaloric and met NRC requirements for normal pregnancy [30]. Amounts of casein hydrolysate (MPH 955; New Zealand Milk Products) and SPI (Soy Clinical Blend IB1.2; total isoflavone content was 3.98 mg/g protein, total aglycone equivalents were 2.32 mg/g protein; DuPont Protein Technologies) added to liquid TEN diets were 31.5 and 31 g/l, respectively. Ethanol was infused at increasing amounts to reach a maximum of 10 g/Kg body weight. At GD 19, ethanol was no longer fed, however, the TEN diets were infused until parturition and simultaneously, corresponding solid diet was added to cages. After parturition, only water was infused (25 ml/23 h) and solid diets were continued *ad libitum*. At PND 2, each litter was culled to 5 males and 5 females (females were used in an unrelated experiment). Progeny were weaned to the same diet as for their dam. Number of male progeny allocated to casein and SPI diets was 24 and 29, respectively.

### Visualization of ACF

Fifteen animals of each diet group in Experiments I and II were used for ACF determination. Colon contents were removed by flushing from the cecal end with ~20 ml of PBS via syringe. Each colon was slid onto a 2 ml pipette and was fixed in this position for 10 min in 10% neutral buffered-formalin. The colon was opened, laid flat and placed between sheets of labeled filter paper in fixative in the cold. Tissue was removed from formalin, divided into proximal and distal halves, and stained in 0.2% methylene blue in PBS for 5–7 min or until the tissue had a uniform blue appearance. Tissues were rinsed with PBS for ~1 min and stored in 0.4% formalin-PBS at 4°C. Aberrant crypt foci were viewed immediately after staining using a Nikon AMZ800 stereoscope at 40× magnification with side illumination. Proximal and distal colon halves were reviewed along their entire lengths and all ACF were counted. Aberrant crypt foci were categorized according to crypt complexity (1, 2, 3, 4, 5, etc., crypts per ACF). All colons were scored in blinded fashion by a single observer. Colons that failed to yield useable data due to poor fixation and/or staining were excluded from statistical analysis.

### TUNEL

Colons not used for ACF determination (i.e., whole-mount fixation) were divided into proximal and distal halves and the midpoints of each half taken for fixation. Tissue was fixed in 10% neutral buffered-formalin, processed through a graded series of ethanol and xylene washes, embedded in paraffin, 4 μm sections obtained, and these were subjected to TUNEL assay. The TdT-FragEL DNA Fragmentation Detection kit (Oncogene Research Products, San Diego, CA) was used for this purpose. Approximately 200 crypt columns were examined from each of 4–5 animals in each diet group.

### Adiposity

Body composition data were obtained on anesthetized rats by dual energy x-ray absorptiometry (DXA) using a Hologic QDR 4500A instrument (Bedford, MA). Five rats (at 33 days post-AOM treatment) were randomly chosen from casein and SPI diet groups (*Expt. I*) for DXA analysis. Percentage of global fat (% fat) was determined.

### Statistical Analyses

The ACF endpoints were discrete variables (numbers of crypts, numbers of ACF, etc.) and hence were not normally distributed. We took the natural logarithm of all data points since log-transformed values were less skewed than the original data and more amenable to analysis. However, for simplicity, figures and tables present the original (not log-transformed) data. To examine diet effects, we used the SAS System's (SAS, SAS Institute, Inc., Cary, NC) PROC GLM procedure. Unpaired t tests were also used to examine differences between proximal and distal colon for several endpoints. Incidence of large ACF (with 5 or more crypts per focus) was analyzed using Fisher's Exact Test (SigmaStat for Windows Version 2.03, SPSS Inc.). For all analyses, a P value less than 0.05 was considered significant, while 0.05 < P < 0.1 was deemed marginal. Body weights and body fat content were compared by t-test (SigmaStat).

## Results

### Effects of Lifetime Consumption of Casein and SPI on Colonic ACF Frequency

**Figure 1:**
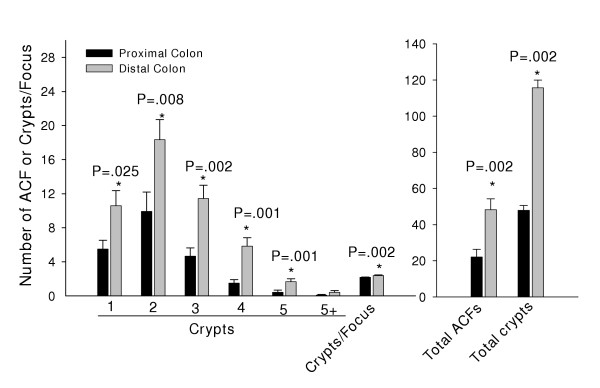
ACF occurrence in proximal and distal colon of Sprague Dawley rats (n = 12) lifetime-fed casein. Shown are means ± SEM of ACF (per rat) containing: 1, 2, 3, 4, 5 or greater than 5 crypts (5+) per ACF; crypt multiplicity (number of aberrant crypts/focus); total number of ACF; and total number of aberrant crypts (no. ACF × crypts/focus). P values indicate all statistically significant differences between proximal and distal colon.

In casein-fed animals, the distal half of the colon had 2–3 fold more ACF of each size class than did the proximal half (Fig. 1). In rats lifetime-fed casein or SPI, differences in ACF content were observed for distal colon (Table 1). SPI feeding led to fewer numbers of ACF with 4, 5 and >5 crypts and as a consequence, a reduced overall ACF crypt multiplicity for distal colon. Crypts/focus was slightly reduced (by ~8%) by SPI in the proximal colon (P = 0.01). SPI did not differ from the casein group in the frequencies of ACF containing 1, 2 or 3 aberrant crypts in either colon region.

**Table 1:** Effect of diet on ACF distribution by colon region*

	Proximal Colon		Distal Colon	
		
	CAS	SPI	CAS	SPI
ACF – 1 Crypt	5.50 ± 1.03	6.55 ± 1.35	10.58 ± 1.77	12.55 ± 2.45
ACF – 2 Crypts	9.92 ± 2.28	8.65 ± 1.61	18.33 ± 2.36	16.27 ± 2.81
ACF – 3 Crypts	4.67 ± 0.96	5.18 ± 1.17	11.42 ± 1.57	8.18 ± 1.91
ACF – 4 Crypts	1.50 ± 0.40 ^a^	0.82 ± 0.38	5.83 ± 0.99 ^b^	2.45 ± 0.73
ACF – 5 Crypts	0.42 ± 0.26	0.27 ± 0.20	1.67 ± 0.33 ^c^	0.64 ± 0.47
ACF – 5+ Crypts	0.08 ± 0.08	0.09 ± 0.09	0.42 ± 0.19 ^d^	0.09 ± 0.09
Crypts/Focus	2.17 ± 0.05 ^a^	2.00 ± 0.09	2.38 ± 0.07 ^c^	2.02 ± 0.04
ACF Total	22.08 ± 4.29	21.54 ± 4.08	48.25 ± 5.98	40.18 ± 6.93
Crypt Total	47.92 ± 9.47	44.55 ± 8.98	115.67 ± 14.83 ^e^	83.18 ± 15.55

* CAS, n = 12; SPI, n = 11; shown are means ± SEM per animal for ACF of differing sizes; 5+ = no. ACFs containing 6 or more aberrant crypts; crypt total = sum of ACF × crypts/focus.

^a^P = 0.01, ^b^P = 0.005, ^c^P = 0.001, ^d^P = 0.091, ^e^P = 0.044; P values for differences between diets within each region; SAS.

### Dietary Switchovers at Weaning and Subsequent ACF Formation

To examine the possibility that dietary exposure to SPI over the period encompassing fetal and neonatal development could mimic effects of lifetime SPI, we performed diet switchovers of Sprague Dawley rats at weaning and surveyed their colons six weeks after AOM administration, relative to animal's lifetime-fed SPI diet concurrently. The opposite switchover, from casein to SPI at weaning, was carried out in parallel to examine effects of SPI, from post-weaning through to adulthood.

**Figure 2:**
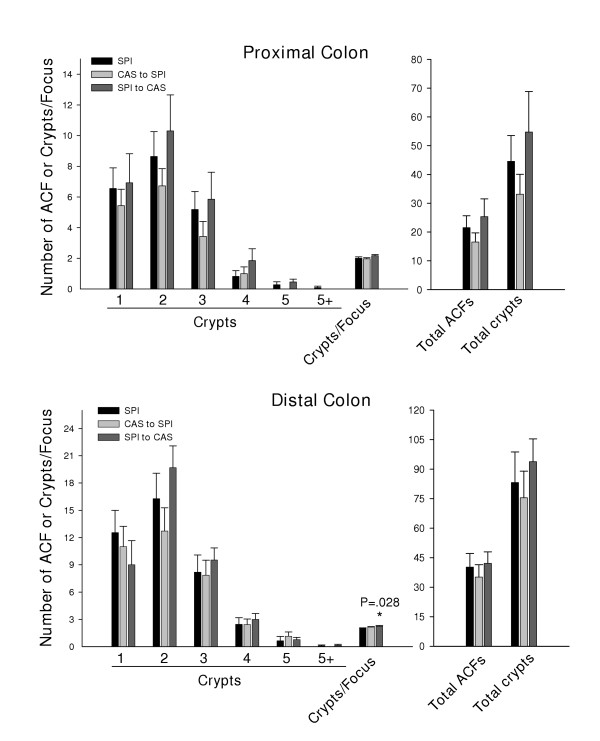
Frequency distribution of ACF in Sprague Dawley rats lifetime-fed SPI or switched, at weaning, from CAS to SPI or from SPI to CAS. Analysis of proximal and distal colon halves is shown. Shown are means ± SEM, per rat, of ACF containing: 1, 2, 3, 4, 5 or greater than 5 crypts (5+) per ACF; crypt multiplicity (number of aberrant crypts/focus); total number of ACF; and total number of aberrant crypts. P value indicates the only statistically significant difference between switchover diets and SPI.

ACF frequency for each diet switchover generally mimicked that for lifetime SPI (Fig. 2). Analysis for the relative incidence rather than the mean number (per animal) of the largest ACF (those containing 5 or >5 crypts) for all diet groups is shown in Table 2. These data further support a protective role for dietary SPI (lifetime, CAS/SPI or SPI/CAS regimens) on appearance of large ACF.

**Table 2:** Relative incidence of largest ACFs (5 or >5 Crypts/ACF)*

	CAS	SPI	CAS/SPI	SPI/CAS
% rats with ACF(s) containing 5 crypts				
Proximal	25	18	0	38
Distal	83	18^a^	57	54
Entire	83	36^b^	57	69
% rats with ACF(s) containing >5 crypts				
Proximal	8	9	0	0
Distal	33	9	0	15
Entire	42	18	0	15

* CAS, n = 12; SPI, n = 11; CAS/SPI, n = 7; SPI/CAS, n = 13.

^a, b^Compared to CAS: ^a^P = 0.003; ^b^P = 0.036; Fisher's Exact Test.

### Apoptosis

At six weeks, post-AOM, there were no observable differences in the relative apoptotic state of total colonic epithelium in distal colons of CAS, SPI, CAS/SPI and SPI/CAS groups (Table 3). However, the upper third crypt region of the CAS/SPI group had ~2-fold more apoptotic cells than did the lifetime SPI or SPI/CAS groups.

**Table 3:** Effect of diet regimen on TUNEL positive cells in the distal colon^a^

	CAS^b^	SPI	CAS/SPI	SPI/CAS
Total^c^	18.0 ± 2.55	13.8 ± 3.20	24.6 ± 5.80	16.0 ± 3.89
Upper^d, e^	6.8 ± 3.02	3.2 ± 1.07	11.4 ± 2.02	2.25 ± 0.48
Middle	4.6 ± 0.93	3.2 ± 1.02	9.2 ± 4.68	9.5 ± 3.12
Lower	6.8 ± 2.63	7.2 ± 1.93	4.4 ± 1.29	4.75 ± 0.75

^a^CAS, n = 5; SPI, n = 5; CAS/SPI, n = 5; SPI/CAS, n = 4 animals; PND 138.

^b^Mean values are expressed as the number of positive cells per 200 crypt columns ± SEM.

^c^Data are for entire crypts.

^d^Data represent upper one-third, middle one-third or lower one-third regions of crypts, respectively.

^e^Overall diet effect (P = 0.024); CAS/SPI > SPI and >SPI/CAS, P < 0.05; One-way ANOVA and Student-Newman-Keuls method.

### Growth and Body Composition

**Figure 3:**
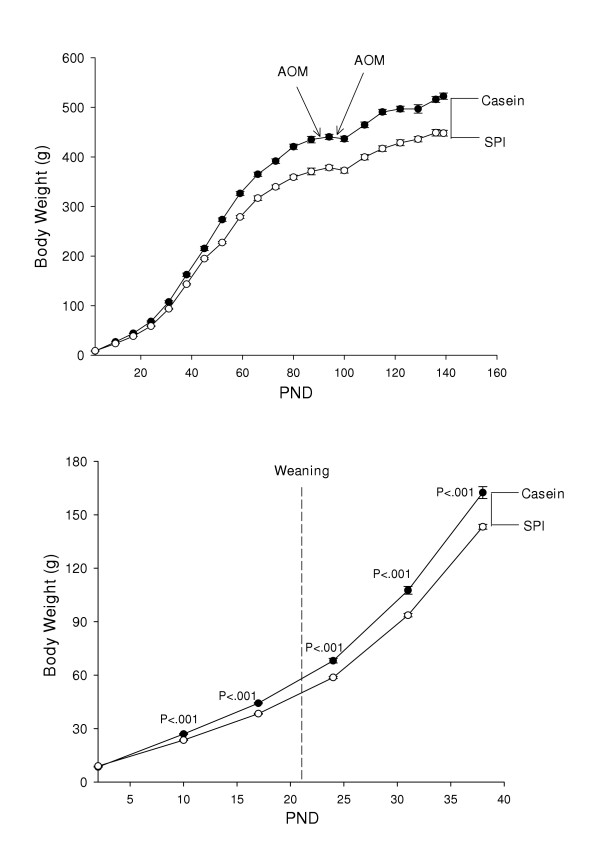
Feeding of casein or SPI to pregnant dams and their progeny elicits differential growth rates. Top panel shows body weight (mean ± SEM) gain in relation to postnatal day (PND) and time of administration of azoxymethane (AOM) for progeny of Sprague Dawley dams. Bottom panel shows divergence in body weights during early postnatal development. P values indicating differences between CAS and SPI groups (t-test) are shown in lower panel.

**Figure 4:**
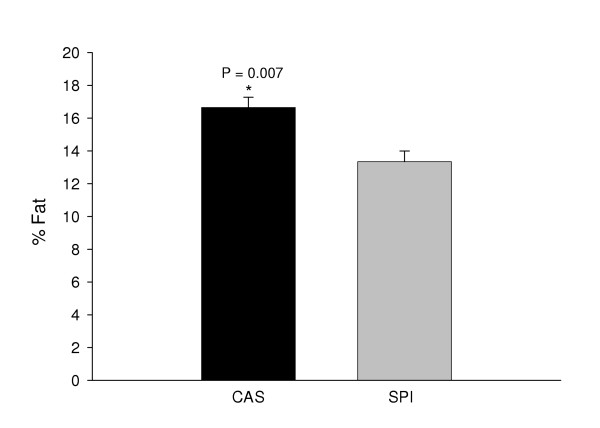
Dietary protein type influences body fat content. DXA was performed on five animals per group (CAS: casein; SPI: soy protein isolate) at 33 days after the second AOM administration. Means (±SEM) are statistically different for the two diets.

**Figure 5:**
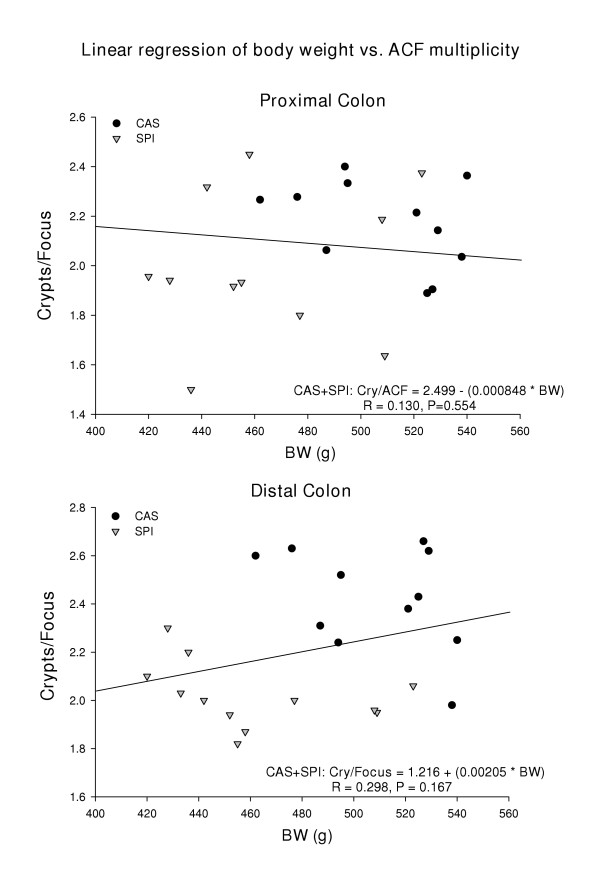
Lack of association of final body weight with ACF size.

SPI-fed animals weighed less than corresponding casein-fed counterparts (Fig. 3). The body weight differences between the casein and SPI groups were evident as early as PND 10 postnatal and prior to weaning (Fig. 3). Administration of AOM resulted in temporary cessation of growth and a small amount of weight loss in all groups (Fig. 3); however after a short lag period, all animals resumed growth. Growth curves of casein/SPI and SPI/casein switch-over groups were intermediate between those for lifetime casein and SPI groups (data not shown). Analysis of body composition (at 33 days post-AOM treatment) by DXA revealed significant differences in global % fat between groups (CAS > SPI, difference of ~3.3 percentage points; Fig. 4) and this somewhat mimicked the observed final differences in body weight. Regression analysis did not identify any significant associations between final body weight and proximal or distal ACF numbers or crypts/focus (Fig. 5 and data not shown).

### ACF in AOM-treated Adult Rats Previously Exposed to Ethanol as Fetuses

**Figure 6:**
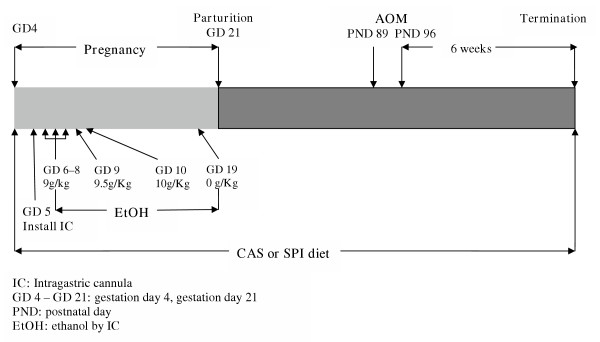
Experimental design used for evaluation of diet effects on ACF in progeny of dams exposed to ethanol during pregnancy.

**Figure 7:**
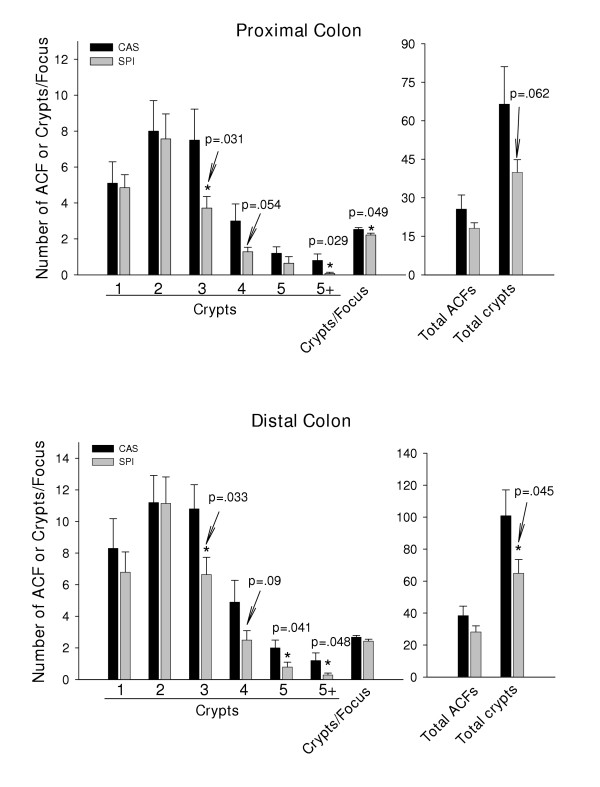
Frequency distribution of ACF in rat progeny lifetime fed casein (n = 10) or SPI (n = 14) and whose dams were exposed to ethanol during pregnancy (Fig. 6). Analysis of proximal and distal colon halves is presented. Shown are mean ± SEM, per rat, of ACF containing: 1, 2, 3, 4, 5 or greater than 5 crypts (5+) per ACF; crypt multiplicity (number of aberrant crypts/focus); total number of ACF; and total number of aberrant crypts. P values indicate statistically significant differences between diets.

We confirmed the inhibitory effect of SPI on occurrence of large ACF in a second model, namely, progeny of Sprague Dawley dams that received ethanol + casein or ethanol + SPI by total enteral nutrition (TEN) during gestation and which were weaned to the same diet as their dams (paradigm shown in Fig. 6). We chose this model since we were interested in examining how diet, in combination with ethanol, might affect ACF distribution in progeny. As is evident from Fig. 7, SPI inhibited the occurrence of large ACF relative to the casein diet in animals exposed to ethanol as fetuses. Unlike the results from Expt. I. however, SPI-fed animals exhibited reduced ACF numbers in both proximal and distal colons and these ACF now included those with 3 aberrant crypts (Fig. 7). Moreover, there was an increased occurrence of ACF with three or more crypts in the proximal colons of casein-fed rats in Expt. II vs. those in Expt. I. In the absence of a true no-ethanol control for Expt. II, we cannot make any final conclusions about the specific effect of fetal alcohol exposure on ACF frequency in the later adult stage. However, these data do suggest that fetal alcohol exposure favors the development of large ACF which was inhibited by SPI in the diet.

## Discussion

The objectives of this study were to: a) elucidate effects of SPI on colon ACF frequency in rat models, b) study effects of dietary SPI on somatic growth and adiposity in view of their potential interactions with colon ACF incidence, c) examine developmental and lifetime 'exposures' to dietary SPI and consequent effects on ACF indices, and d) correlate ACF data with our previously published colon tumor data obtained with animals lifetime fed SPI [21]. ACF may provide a more time- and cost-effective means to study SPI effects on colon cancer prevention in animal models and we sought to further explore this possibility in the current study. We report that SPI reduced the incidence of the largest size classes of ACF in AOM-treated, adult Sprague Dawley male rats and, regardless of whether SPI was fed during GD 4 – PND 21, only after PND 21, or from GD 4 to PND 138. This effect of SPI also was manifested in animals whose dams were exposed to ethanol during pregnancy; thereby suggesting the generality of the SPI effect.

ACF have been identified on the colon luminal surface in rats and mice treated with chemical carcinogens (AOM, DMH, and NMU) and in humans with and without overt colon carcinoma [31,32]. AOM stimulates both proliferation and apoptosis in the colonic mucosa [33] and ACF are thought to be the earliest observable pre-neoplastic lesions to arise in this tissue [34,35]. The validity of ACF as an intermediate biomarker for colon cancer is however, controversial. Larger multi-cryptal ACF are generally correlated with tumor incidence in rodents and humans [32,36-42]. Feeding a Western-type diet can induce ACF as well as adenomas and carcinomas in normal mouse colon [43]. Thiagarajan *et al*. [16] reported a trend for diets that promoted ACF numbers to also increase colon tumor numbers. In other studies, however, DMH or AOM induced the occurrence of adenomas and adenocarcinomas in the distal colon which were correlated with ACF number; whereas the proximal colon developed signet-ring type carcinomas which were not correlated with ACF [44-46]. Moreover, there may be discrete subset(s) of ACF, not easily recognized by the standard assay, that progress to microadenomas and which are characterized by increased dysplasia, altered oncoprotein and tumor suppressor expression and/or genomic instability [37,47-51]. Previously, we reported that Sprague Dawley rats, lifetime fed casein-AIN-93G diet had a 50% incidence, whereas those fed SPI-AIN-93 G diet had a 12% incidence of colon tumors, at 40 weeks post-AOM [21]. In our previous study, tumor incidence was similarly reduced for proximal and distal regions by SPI. However, in the present study, we found that SPI reduced crypt multiplicity and frequency of large ACF (those containing 4 or more crypts) mainly in the distal colon, which at six weeks post-AOM had more ACF of each size than did the proximal region. Therefore, the present ACF data are in general concordance with our previous tumor data but only for the distal colon. The basis for the discordance of ACF and tumor data for the proximal colon is unknown but is in general agreement with other studies showing correlations of larger ACFs with distal but not proximal colon tumors [44-46].

A diet switching paradigm examined whether the suppressive effect of SPI on larger ACF required lifetime exposure or could be mimicked with a shorter developmental period of SPI feeding. We also questioned if 'protective' effects could be transferred maternally to the offspring. The results of this experiment are of interest for a number of reasons. The observation that SPI elicited reduced growth by day 10, postnatal, may point to a 'maternal effect' of SPI, during gestation or lactation or both that is transmitted to progeny to affect their growth. The exact nature of this effect awaits further clarification. Both diet switchovers generally mimicked the effects of lifetime SPI on ACF incidence, although the casein/SPI regimen appeared to be slightly more effective than the SPI/casein dietary treatment in this regard. We therefore surmise that SPI can manifest long lasting effects on ACF incidence and that at least some of these effects can be transmitted to the offspring through feeding of SPI to pregnant and/or lactating dams.

Obesity is considered by some to be a promoter of oncogenesis in rat models of chemically induced colon cancer [52,53] and of colon cancer in humans [54]. Positive interactions of soyfoods with increasing body mass index to reduce breast cancer risk in humans have been suggested [55]. In the current study, DXA analysis identified differences in global % fat that could account for the differences between final body weights of casein and SPI animals; although we examined only a limited number of animals in this regard. However, we were unable to find any statistically significant associations of final body weight and ACF for the diet groups. Therefore, our results do not indicate any obvious positive relationship between relative fat content and ACF incidence.

Kállay et al. [56] observed that the feeding of soy protein isolate selectively suppressed or enhanced colonic gene expression (relative to casein). Their work as well as the present results underscores the need for further elucidating direct and indirect actions of dietary SPI and its protein and non-protein constituents on colonic mucosal growth and differentiation during development through to adulthood. The somewhat conflicting literature regarding effects of soy on colon carcinoma in the rat may derive in part from differences in the nature of the dietary casein and SPI used, and the relative developmental timing of SPI feeding. Studies that used commercial diets containing soy constituents for propagation of rat colonies for subsequent experiments may also have been confounded by pre-exposure to soy. It may be important to standardize this developmental soy exposure in future studies so as to reach consensus on the colon cancer-preventive actions of soy and its constituent(s).

## Conclusions

SPI inhibited the development of large ACF, some of which may be tumor precursors. Feeding of SPI to rat dams during pregnancy and lactation led to a suppression in numbers of large ACF in their progeny, implying a long-term or permanent anti-carcinogenic effect elicited by the SPI-based diet. Body weight and body composition were differentially affected by soy protein isolate or casein in the diet. ACF may be a valid intermediate endpoint for elucidating effects of SPI and its biochemical constituents in tumor prevention in the colons of Sprague Dawley rats.

## Author's contributions

ALL performed the whole-mount and TUNEL assays, performed data analysis, and assisted with tissue collection. RX performed data analysis, assisted with tissue collection, and prepared the final figures. JGP and PMS performed statistical analyses. TMB participated in the design of the study and oversaw the animal and diet manipulations. FAS conceived of the study, participated in its design, interpreted the data and prepared the manuscript. All authors read, modified and approved the final manuscript.
